# Arthroscopic Excision of an Intra-Articular Osteochondroma of the Knee

**DOI:** 10.7759/cureus.17154

**Published:** 2021-08-13

**Authors:** Vasileios Chouliaras, Ilias Sotiriadis, Dimitrios A Flevas

**Affiliations:** 1 Orthopaedic Surgery, General Hospital of Arta, Arta, GRC; 2 Arthroscopy and Orthopaedic Surgery, Metropolitan Hospital, Athens, GRC

**Keywords:** ostechondroma, knee, arthroscopy, benign tumor, arthroscopic resection

## Abstract

Osteochondroma is the most common benign tumor of the growing bone that commonly involves the knee joint region. Hereby we present a case of an arthroscopic resection of a symptomatic intra-articular osteochondroma of the knee. A 32-year-old woman presented with diffuse and persistent pain of her right knee followed by discomfort for over three months. She did not report any history of injury or any other medical problem. Physical examination and imaging, with plain radiographs and MRI, revealed a bony mass arising from the supero-lateral aspect of her right distal femur without a stalk. This bone tumor, an osteochondroma, was arthroscopically resected and the diagnosis was confirmed by the histologic examination. The arthroscopic resection of this benign tumor led to complete relief of the symptoms of the patient and her return to daily and athletic activities in one month postoperatively. No recurrence of symptoms occurred during the seven-year follow-up period. Arthroscopic resection of a symptomatic osteochondroma is less painful, more cosmetically accepted, and can result in a quicker recovery than the traditional approach with an open incision.

## Introduction

Osteochondroma constitutes the most common benign bone tumor [1-2]. Usually, it arises from the metaphyseal region of the growing skeleton around the knee and proximal femur [3]. It consists of trabecular bone covered by a cartilage cap [4]. Solitary osteochondroma appears as a cartilage-capped osseous projection featured from the surface of the bone. The tumor usually is located extra-articularly mainly in the proximal humerus, the distal femur, or the proximal tibia [1-2, 5]. Osteochondroma is usually asymptomatic and when clinical symptoms are present these might be as a result of traumatic contusion or pressure on adjacent muscles, joints, nerves, or blood vessels [6]. In cases involving the knee joint, the ostoechondromas are usually para-articular [1]. When the osteochondroma is located intra-articularly usually it causes symptoms such as pain, synovitis, or locking depending on the exact localization [2, 6-7]. Other symptoms that may be present are fracture of the lesion itself and inflammatory changes of the bursa exostotica covering the cartilaginous cap [6].

Hereby we present a case of a female patient who presented with a solitary intra-articular osteochondroma that was treated arthroscopically.

## Case presentation

A 32-year-old female patient presented with a history of persistent right knee pain for over three months. There was and no history of any kind of trauma. Her medical history revealed no other medical conditions. Physical examination did not reveal any knee signs. The range of motion was normal although the patient had mild pain in excessive flexion (over 120°). The symptoms of the patient were mimicking blocking symptoms due to mechanical causes at excessive flexion and full extension. She also described an occasional audible clicking at the superolateral patellofemoral joint. The only remarkable finding was a palpable bony mass over the superolateral aspect of the right knee with tenderness and mild swelling in this area. This lump was hard as a bone, was immobile, and particularly sensitive to palpation. 

**Figure 1:**
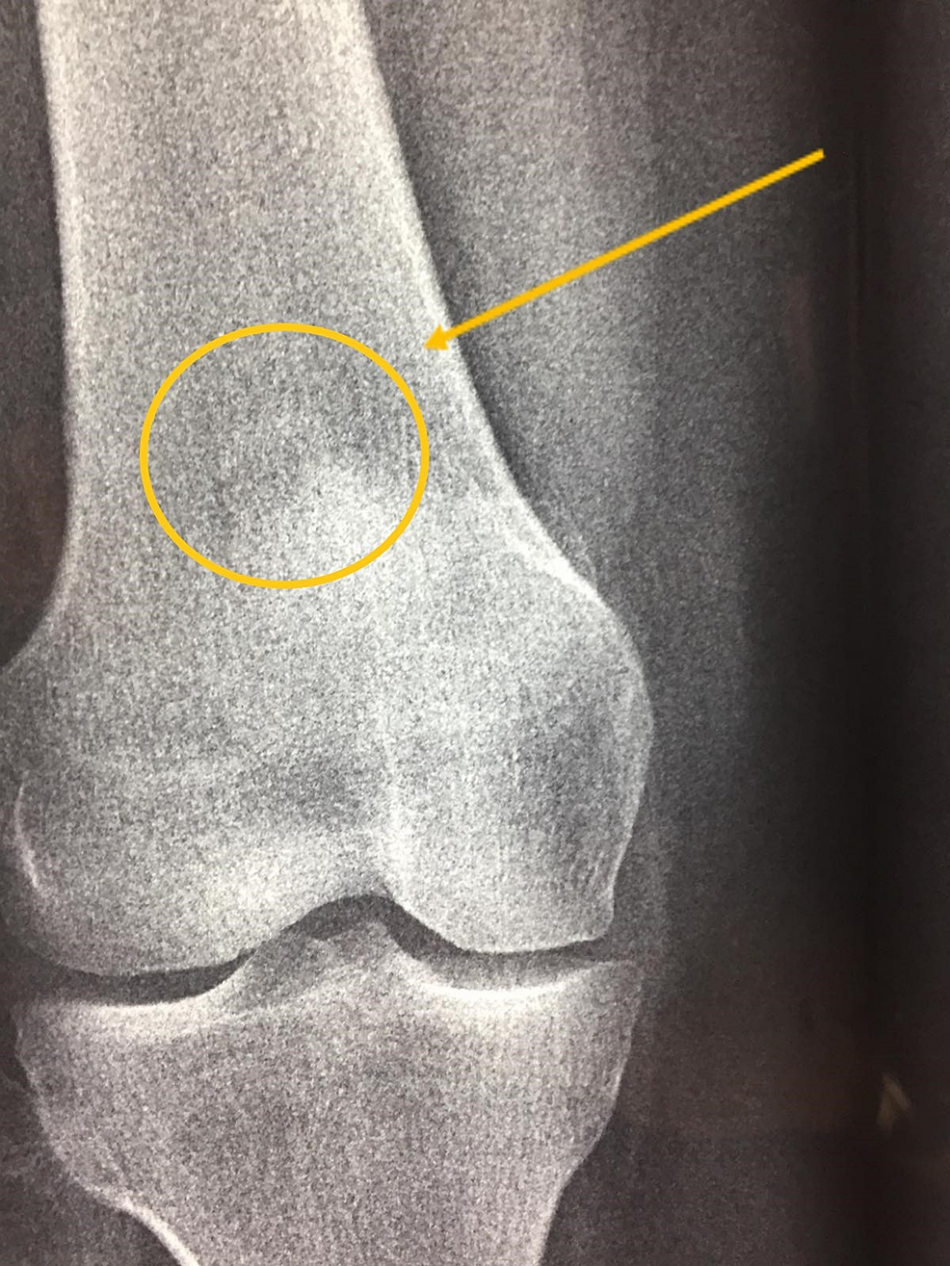
Anteroposterior radiograph of the patient's knee showing the bone lesion (yellow circle and arrow).

**Figure 2:**
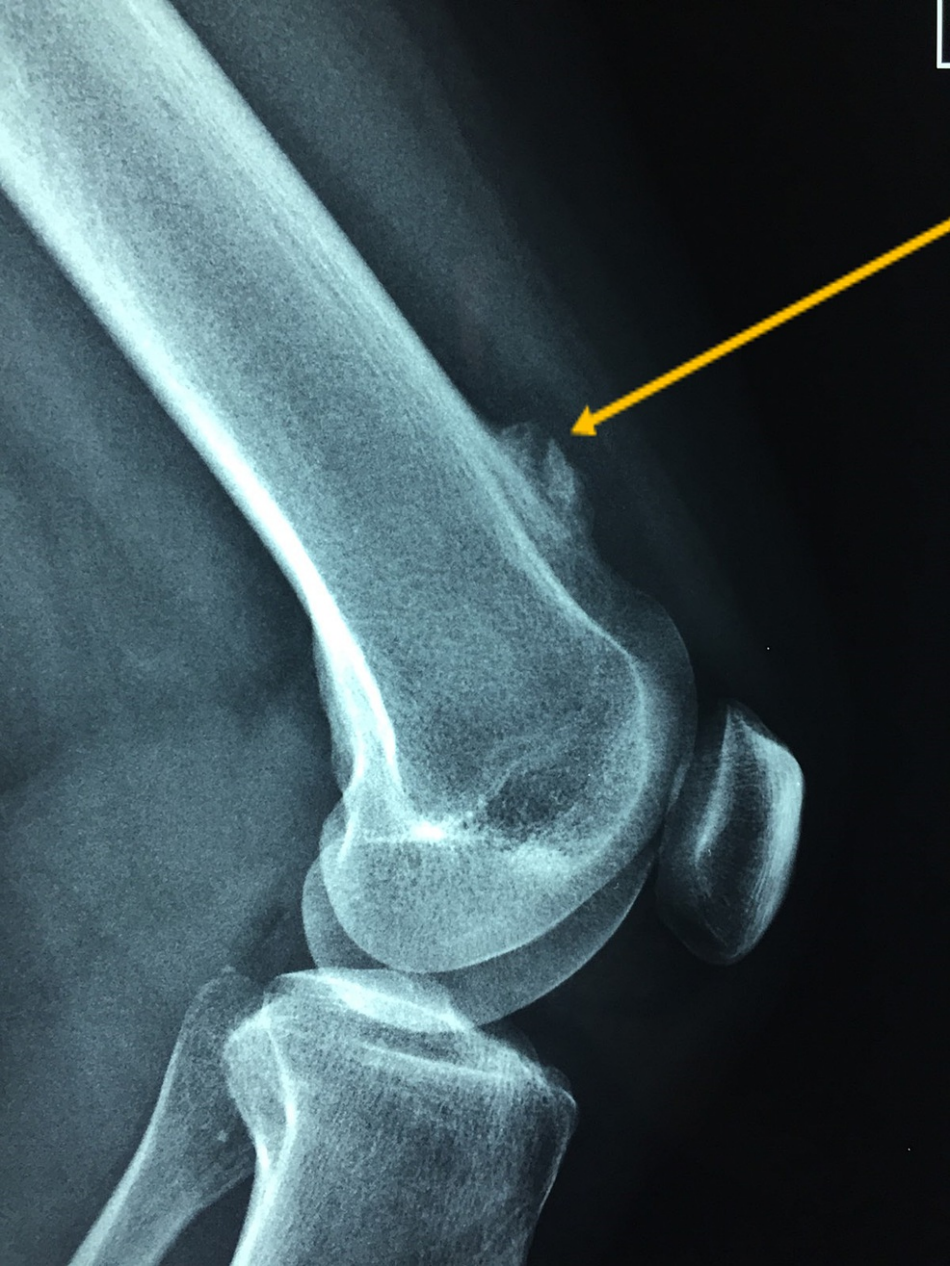
Lateral radiograph of the patient's knee where the bony mass can be seen (yellow arrow).

**Figure 3:**
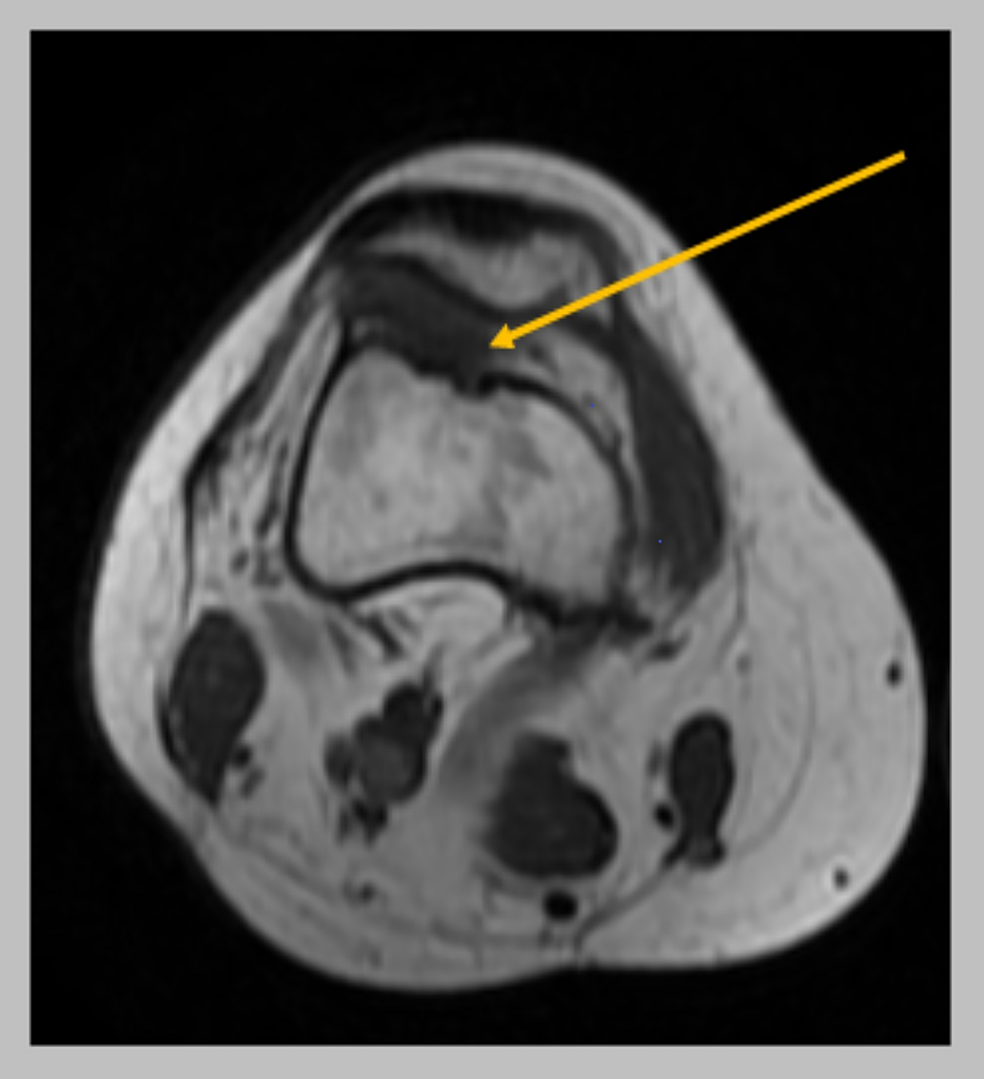
MRI transverse plane showing the lesion (yellow arrow).

**Figure 4:**
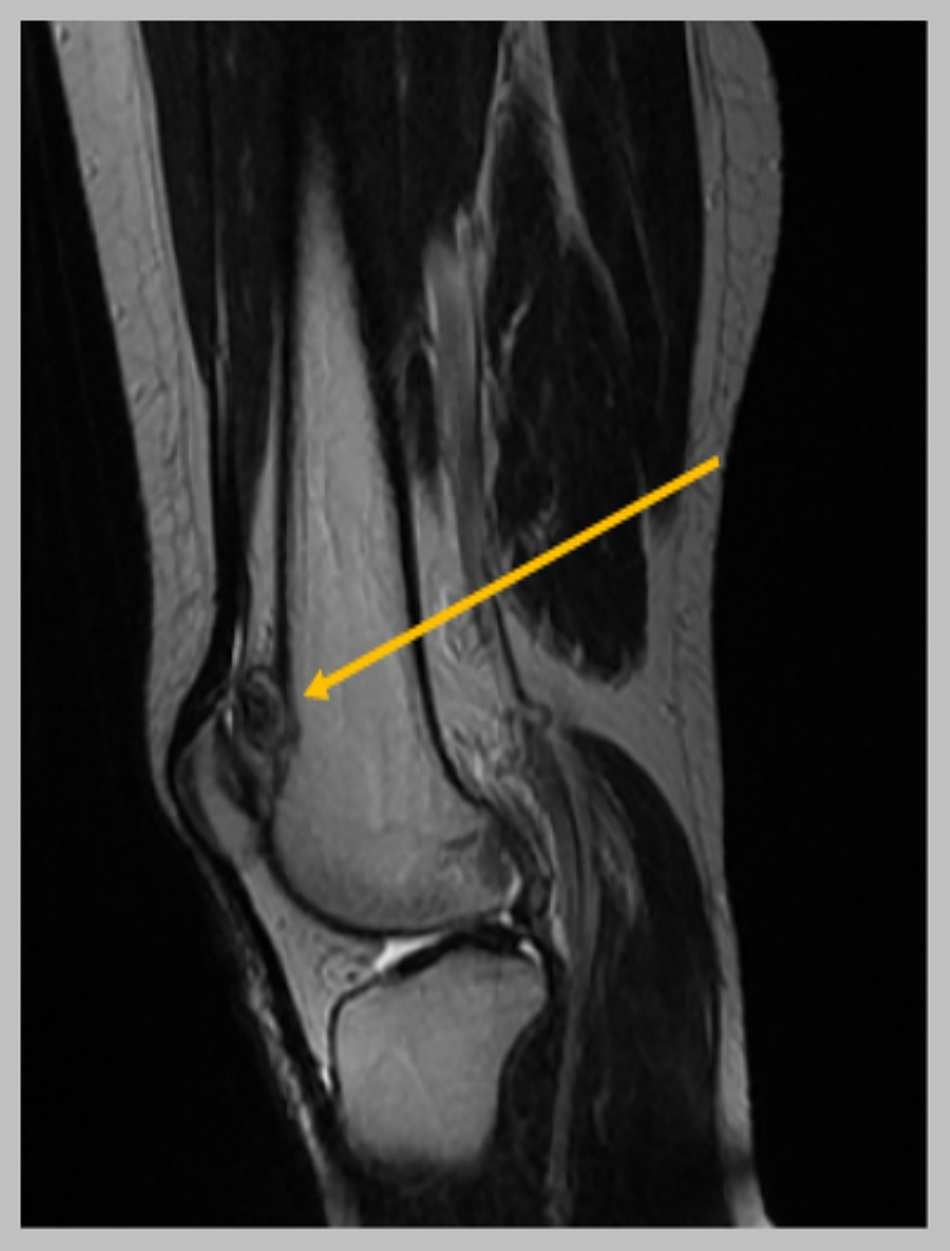
MRI sagittal plane showing the lesion (yellow arrow). It can be seen that the lesion is arising from the femoral bone, on the superior edge of the patellofemoral joint.

**Figure 5:**
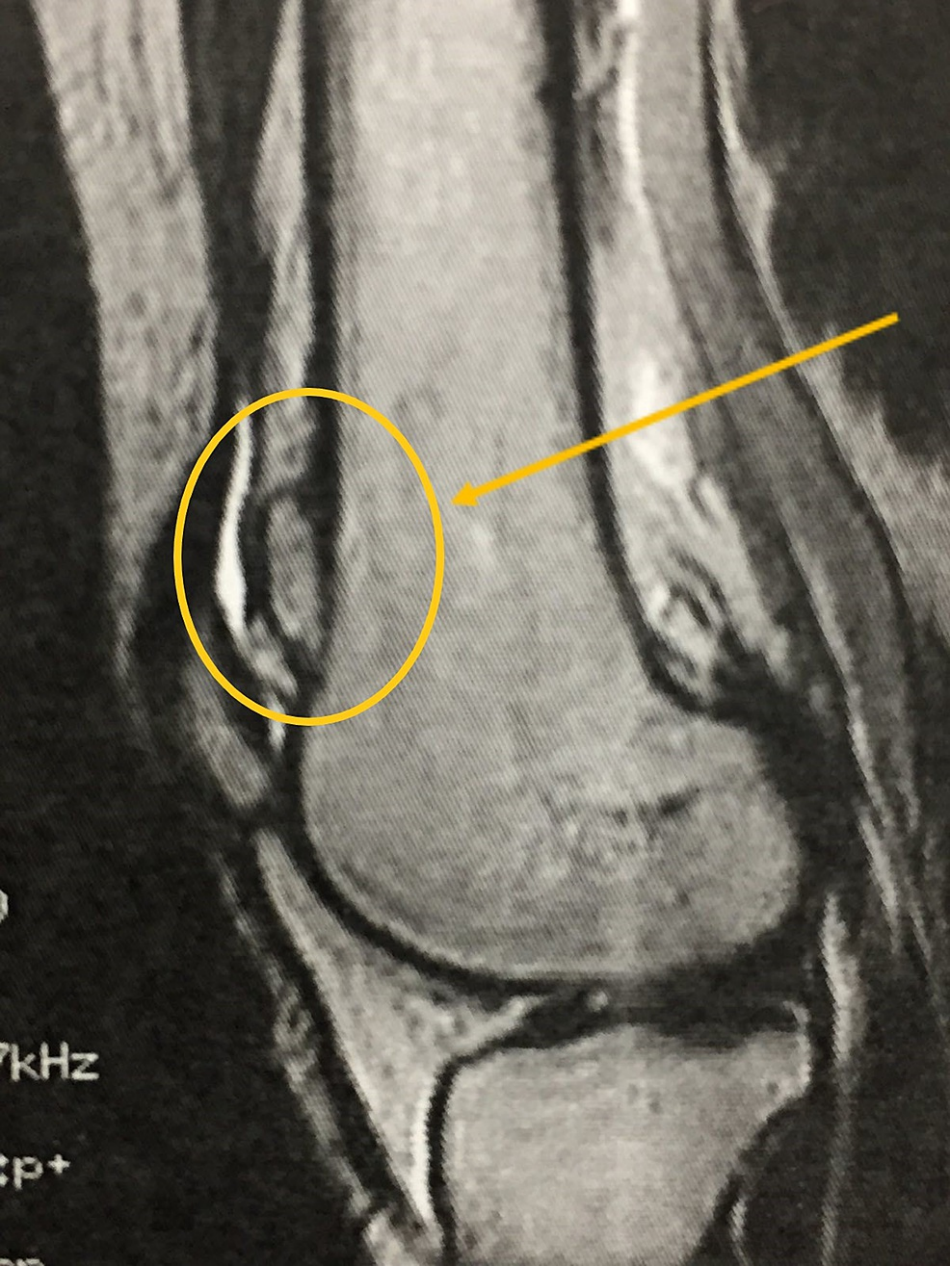
Enhanced image of the MRI sagittal plane where the bone lesion can be seen arising from the femoral bone (yellow circle and arrow).

Plain radiographs revealed a sessile bony mass located at the superolateral aspect of the knee joint without a stalk (Figures 1-2). MRI scan was performed and showed a bony bulge arising from the superolateral aspect of the knee joint (within the joint capsule) with surrounding synovitis (Figures 3-5). The imaging findings and the physical examination (especially the palpable bony mass) posed the diagnosis of solitary intra-articular osteochondroma. Due to the symptoms of the patient, diffuse and persistent pain for over three months, arthroscopic removal of the tumor was decided.

Under general anesthesia, standard arthroscopic portals were performed and the arthroscopic examination of ligaments, menisci, and cartilage was normal. Τhen with the aid of a needle the tumor was marked in the superolateral aspect of the knee joint, surrounded by a bursa, and superomedial and superolateral portals were performed.

**Figure 6:**
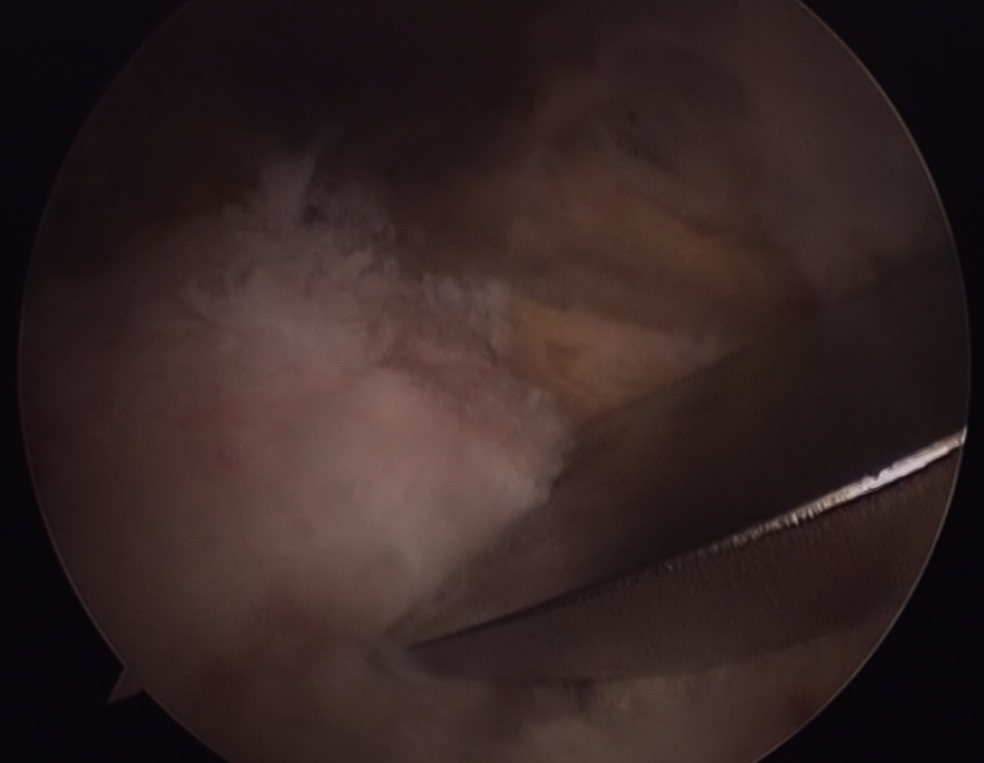
Arthroscopic image of the removal of the osteochondral lesion with an osteotome.

The bursa was removed with the shaver and from the superolateral portal an osteotome was inserted at the base of the tumor (Figure 6). With precise movements, the tumor was excised and finally removed with a hemostatic clamp through the portal. Finally, a burr shaver was used to abrade the bone of the femur.

The histopathological report revealed signs that confirmed the diagnosis of osteochondral lesion.

**Figure 7:**
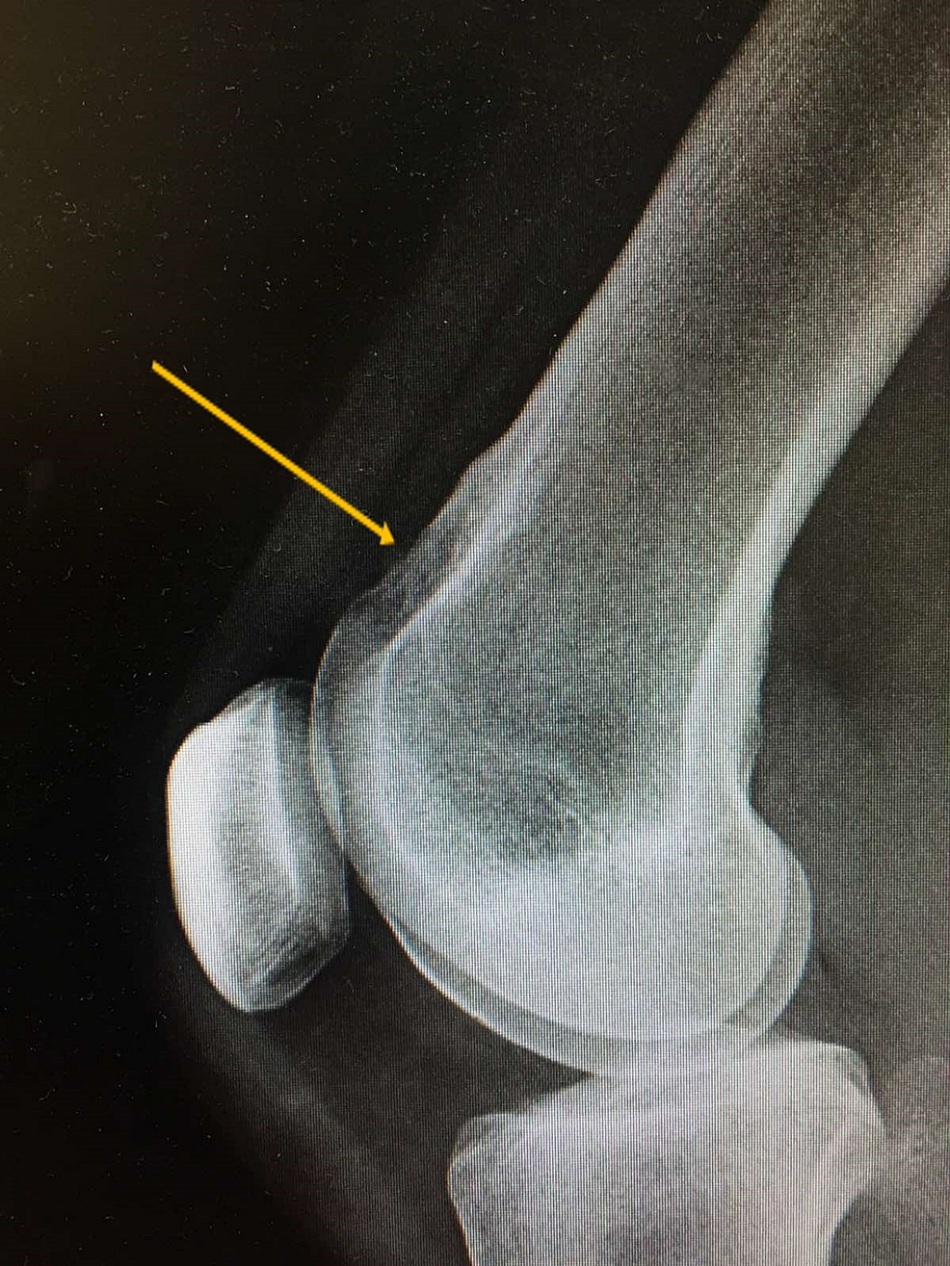
Lateral radiograph of the patient's knee six months postoperatively showing complete removal of the bone lesion (yellow arrow).

The patient started immediately weight bearing from the first postoperative day and followed an aggressive rehabilitation program with muscle strengthening. One month postoperatively the patient claimed complete relief of her symptoms and returned to her full daily activities. Radiographs at six months postoperatively showed no signs of the bone lesion (Figure 7). On a follow-up seven years postoperatively, the patient remains free of symptoms without any recurrence of the tumor.

## Discussion

Osteochondroma which is also known as osteocartilaginous exostosis, is reported to be the most common benign tumor of the bones [4]. More specifically, extra-articular osteochondromas are the benign tumors, solitary or multiple, that are most commonly presented during childhood and adolescence, while intra-articular osteochondromas are seen very rarely [1]. In patients who are skeletally immature, osteochondromas occur around the growth plate of long bones and move towards the diaphysis with the connected bone. Therefore, it is rare for osteochondromas to be located within the articular compartment of a joint in adults [2, 8]. There have been genetic studies that identified a strong association between hereditary multiple osteochondromas and the loci of exostosin-1, 2 and 3 (chromosome 8q24.1, chromosome11p13 and short arm chromosome 19 respectively) [1, 9]. Regarding symptoms, for both extra-articular and intra-articular lesions, there have been reported pain, discomfort, and restriction of the joint motion. The precise pathogenetic mechanism of these tumors remains unknown. However, cartilaginous metaplasia of articular and para-articular connective tissue seems to be the main cause [8].

Solitary osteochondromas may be sessile or pedunculated. The tumor might be like a cauliflower or it might be flat, tubular, or hemispheric. Usually, a bursa may be developed over the tumor and covers the osteochondroma. This bursa leads to local synovitis which might be the cause of pain and of the restriction in the free range of motion.

In plain radiographs, the osteochondroma has typical features. The tumor protrudes from the host bone either as sessile or penduculated, and additionally the cortex and cancellous bone of the osteochondroma blends with the cortex and cancellous bone of the host. Because of this typical radiographic appearance, the osteochondroma usually is easily diagnosed based on the plain radiographs. However, imaging which includes preoperative X-rays and MRI scanning is essential for diagnosis, especially when its findings are combined with medical records and clinical symptoms. Differential diagnosis should include synovial chondromatosis, low-grade chondrosarcoma, and osteosarcoma [1, 9-11].

Because of its benign nature, solitary osteochondroma does not need to be surgically excised if it is asymptomatic. Surgical resection is the choice of treatment for symptomatic cases. Usually, the main symptom is diffuse pain (depending on the location of the tumor), or if the tumor is intra-articular it appears with symptoms involving the range of motion of the affected joint. An arthroscopic resection is a treatment option depending on the size and the localization of the tumor. The arthroscopic resection is less invasive, leads to better cosmetic results, and additionally is faster in recovery.

Usually, this type of tumor is seen in extra-articular areas, however in our patient, the osteochondroma located intra-articularly caused mild mechanical symptoms. Furthermore, it could have caused degenerative osteoarthritis of the patellofemoral joint of the knee due to its proximity to that joint and the surrounding cartilage chondral surfaces. Although there are reports regarding intra-articular osteochondromas of the hip [12] and ankle [13] joints, to the best of our knowledge, there are limited reports in the literature concerning intra-articular knee osteochondroma resection using arthroscopic techniques.

## Conclusions

The intra-articular solitary osteochondroma is not a common entity. When the localization of this tumor is the knee joint the arthroscopic resection might be the treatment of choice in symptomatic patients. Arthroscopic resection in symptomatic patients is less painful, more cosmetically accepted, especially in young patients, and leads to faster recovery than the traditional open approach.

## References

[1] Tsakotos G, Tokis A, Vlasis K, Demesticha T, Skandalakis P, Filippou D, Piagkou M (2019). Arthroscopic resection of extra-articular knee osteochondroma: report of two cases. J Surg Case Rep.

[2] Kim JI, Kwon JH, Park YJ, D'Almeida VR, Soni SM, Nha KW (2013). Arthroscopic excision of solitary intra-articular osteochondroma of the knee. Knee Surg Relat Res.

[3] Aydin N, Gokkus K, Topal C, Aydin AT (2012). Solitary synovial osteochondroma of the knee: mimicking a giant loose body. Int Med Case Rep J.

[4] Kesgin E, Çelik C, Karaoğlu S (2013). Arthroscopic resection of osteochondroma of the knee: two case reports. Eklem Hastalik Cerrahisi.

[5] Essadki B, Moujtahid M, Lamine A, Fikry T, Essadki O, Zryouil B (2000). [Solitary osteochondroma of the limbs. Clinical review of 76 cases and pathogenic hypothesis]. Acta Orthop Belg.

[6] Takahashi M, Nishihara A, Ohishi T, Shiga K, Yamamoto K, Nagano A (2004). Arthroscopic resection of an intra-articular osteochondroma of the knee in the patient with multiple osteochondromatosis. Arthroscopy.

[7] Schmoyer S, Ciullo JV (2001). Arthroscopic resection of an osteochondroma of the knee. Arthroscopy.

[8] Rizzello G, Franceschi F, Meloni MC, Cristi E, Barnaba SA, Rabitti C, Denaro V (2007). Para-articular osteochondroma of the knee. Arthroscopy.

[9] Porter DE, Lonie L, Fraser M, Dobson-Stone C, Porter JR, Monaco AP, Simpson AH (2004). Severity of disease and risk of malignant change in hereditary multiple exostoses. A genotype-phenotype study. J Bone Joint Surg Br.

[10] Ruan W, Cao L, Chen Z, Kong M, Bi Q (2018). Novel exostosin-2 mutation identified in a Chinese family with hereditary multiple osteochondroma. Oncol Lett.

[11] Stieber JR, Dormans JP (2005). Manifestations of hereditary multiple exostoses. J Am Acad Orthop Surg.

[12] Siebenrock KA, Ganz R (2002). Osteochondroma of the femoral neck. Clin Orthop Relat Res.

[13] Yamashita T, Sakamoto N, Ishikawa I, Usui M, Fujisawa Y (1998). Intra-articular osteochondroma of the ankle joint. J Foot Ankle Surg.

